# Supplementary material for: Questioning the proverb ‘more haste, less speed’: classic versus metabarcoding approaches for the diet study of a remote island endemic gecko
Source: PeerJ. 2020 Jan 2;8:e8084. doi: 10.7717/peerj.8084 (PMC6942681; doi:10.7717/peerj.8084)
Supplement: Table S1 — Fragment length (in base pairs, bp), sequences, and reference of forward and reverse primers are detailed. [file peerj-08-8084-s001.docx]

| **Primer Name** | **Fragment length (bp)** | **Primer F** | **Primer R** | **Reference** |
| --- | --- | --- | --- | --- |
| **12sv5** | 73–110 | 12sv5F | 12sv5R | (Riaz et al. 2011) |
|  |  | TAGAACAGGCTCCTCTAG | TTAGATACCCCACTATGC |  |
| **IN16STK-mod** | ~ 110 | IN16STK-1F-mod | IN16STK-1R-mod | This manuscript  (based on Kartzinel & Pringle (2015)) |
|  |  | TRAACTCAGATCATGTAA | TTAGGGATAACAGCGTWA |  |
| **g/h** | 10–143 | g_F | h_R | (Taberlet et al. 2007) |
|  |  | GGGCAATCCTGAGCCAA | CCATTGAGTCTCTGCACCTATC |  |
| **e/f** | ~146 | e_F | f_R | (Taberlet et al. 1991) |
|  |  | GGTTCAAGTCCCTCTATCCC | ATTTGAACTGGTGACACGAG |  |
