# Supplementary material for: Questioning the proverb ‘more haste, less speed’: classic versus metabarcoding approaches for the diet study of a remote island endemic gecko
Source: PeerJ. 2020 Jan 2;8:e8084. doi: 10.7717/peerj.8084 (PMC6942681; doi:10.7717/peerj.8084)
Supplement: Table S2 — The respective volumes for the different primer sets are provided. [file peerj-08-8084-s002.docx]

| **Reagents (μL)** | **12sv5** | **IN16STK-mod** | **g/h** | **e/f** |
| --- | --- | --- | --- | --- |
| QIAGEN multiplex PCR master mix | 10.4 | 10.4 | 10.4 | 4 |
| Forward primer (10 μM) | 0.4 | 0.4 | 0.4 | 1 |
| Reverse primer (10 μM) | 0.4 | 0.4 | 0.4 | 1 |
| Blocking primer (10 μM) | 8 | - | - | - |
| BSA (20 mg/mL) | - | - | - | 0.5 |
| Ultra-pure water | 2.8 | 10.8 | 10.8 | 16 |
| DNA sample | 3 | 3 | 3 | 3 |
| Total | 25 | 25 | 25 | 25 |
