# Supplementary material for: Questioning the proverb ‘more haste, less speed’: classic versus metabarcoding approaches for the diet study of a remote island endemic gecko
Source: PeerJ. 2020 Jan 2;8:e8084. doi: 10.7717/peerj.8084 (PMC6942681; doi:10.7717/peerj.8084)
Supplement: Table S3 — The temperature (T), time (t), and number of cycles (NC) for each primer set is detailed. [file peerj-08-8084-s003.docx]

| **12sv5** | | |  | **IN16STK-mod** | | |  | **g/h** | | |
| --- | --- | --- | --- | --- | --- | --- | --- | --- | --- | --- |
| **T(ᵒC)** | **t** | **NC** |  | **T(ᵒC)** | **t** | **NC** |  | **T(ᵒC)** | **t** | **NC** |
| 95 | 15' | - |  | 95 | 15' | - |  | 95 | 15' | - |
| 95 | 30" |  |  | 95 | 30" |  |  | 95 | 30" |  |
| 52 | 30" | 40x |  | 45 | 30" | 40x |  | 52 | 30" | 40x |
| 72 | 30" |  |  | 72 | 30" |  |  | 72 | 30" |  |
| 72 | 10' | - |  | 72 | 10' | - |  | 72 | 10' | - |
